# Assessing Hypoendemic Onchocerciasis in *Loa loa* Endemic Areas of Southeast Nigeria

**DOI:** 10.4269/ajtmh.20-0368

**Published:** 2020-09-21

**Authors:** Lindsay J. Rakers, Emmanuel Emukah, Barminas Kahansim, Bertram E. B. Nwoke, Emmanuel S. Miri, Emily Griswold, Emmanuel Davies, Cephas Ityonzughul, Chukwuma Anyaike, Perpetual Agbi, Frank O. Richards

**Affiliations:** 1 The Carter Center, Atlanta, Georgia;; 2 The Carter Center, Owerri, Nigeria;; 3 The Carter Center, Jos, Nigeria;; 4 Parasitology Department, Imo State University, Owerri, Nigeria;; 5 Federal Ministry of Health, Abuja, Nigeria

## Abstract

Nigeria changed its goal for onchocerciasis from control to transmission elimination. Under the control program, ivermectin mass drug administration (MDA) focused only on hyper/meso-endemic local government areas (LGAs) identified by Rapid Epidemiological Mapping of Onchocerciasis as having ≥ 20% nodule rates. Because transmission is likely in some LGAs where nodule rates are < 20%, the new elimination paradigm requires MDA expansion. Determining which hypoendemic areas require MDA, termed onchocerciasis elimination mapping, is a major challenge. In 2016, we studied 19 ivermectin-naive hypoendemic LGAs in southern Nigeria that bordered LGAs under MDA. Fifty adults and 50 children (aged 5–10 years) were tested in 110 villages for onchocerciasis IgG4 antibody using an Ov16 rapid diagnostic test (RDT). A 10% subset of subjects provided a blood spot for confirmatory Ov16 ELISA. The mean prevalence of RDT positives was 0.5% in the 5,276 children tested (village range, 0.0–4.0%) versus 3.3% in 5,302 adults (village range, 0.0–58.0%). There was 99.3% agreement between the Ov16 RDT and ELISA. Six different MDA launch thresholds were applied to the RDT results based on different recommendations by the Nigeria Onchocerciasis Elimination Committee and the Onchocerciasis Technical Advisory Subgroup of the WHO. Mass drug administration targets for the same area varied tenfold by threshold chosen, from one LGA (population to be treated 221,935) to 13 LGAs (population 2,426,987). Because the Ov16 threshold selected will have considerable cost and resource implications, the decision to initiate MDA should incorporate entomological data demonstrating onchocerciasis transmission.

## INTRODUCTION

The parasitic infection onchocerciasis has a devastating impact on quality of life. Spread by repeated bites of black flies of *Simulium* species, *Onchocerca volvulus* larvae can mature into adult worms that group together to form nodules under the skin, from which fertilized females release embryos (microfilariae, or “mf”), which cause eye disease that can lead to blindness and skin disease with maddening itching. Because the black fly vector breeds near well-oxygenated fast-flowing water, the disease is often colloquially referred to as “river blindness.” Nigeria has the greatest number of onchocerciasis cases among the 31 endemic countries.^1–4^

Nigeria has also distributed more Mectizan^®^ (ivermectin; Merck & Co., Inc., Kenilworth, NJ) tablets to prevent onchocerciasis than any other country, with roughly 719 million treatments administered in endemic areas between 1990 and 2018 (personal communication, Perpetual Agbi, Head of Monitoring and Evaluation, Federal Ministry of Health [FMOH]). Ivermectin is donated by Merck & Co. through the Mectizan Donation Program,^5^ which is one of the global partners in the effort to eliminate onchocerciasis. Other partners include the endemic countries, nongovernmental organizations, donors, universities, and the WHO.

A dose of ivermectin will kill the mf and suppress their production by female worms for 4–6 months. The medicine does not kill the adult worms, which can live in nodules for up to 15 years.^5,6^ Good ivermectin treatment coverage for the duration of the life span of the adult worms can ultimately put a stop to transmission of the infection when the worm population is reduced below a critical threshold. Transmission elimination has been demonstrated in four countries in the Americas that have received WHO verification of elimination, as well as parts of Africa which have successfully maintained transmission interruption status for at least 3 years after halting ivermectin mass drug administration (MDA).^7–9^

Rapid Epidemiological Mapping of Onchocerciasis in Nigeria was carried out between 1994 and 1996 using the presence of skin nodules to determine where ivermectin MDA should be launched to control morbidity due to onchocerciasis.^10,11^ In a sample of villages located close to rapidly flowing rivers and streams, a convenience sample of 50 resident adult males were examined for the characteristic palpable onchocercal nodules. If ≥ 40% of the sample had palpable nodules, that area was deemed hyperendemic for onchocerciasis and in urgent need of ivermectin MDA. If ≥ 20 and < 40% had palpable nodules, the local government area (LGA) was deemed meso-endemic, and MDA was deemed desirable. If less than 20% of males in the sample presented with nodules, the LGA was deemed hypoendemic and not eligible for treatment because the risk of eye and skin disease from onchocerciasis was considered minimal.^12,13^ These thresholds were determined by the onchocerciasis control approach that was in place at that time. However, many hypoendemic LGAs were contiguous to areas that were meso- or hyperendemic and could have been harboring transmission. When Nigeria moved to a goal of transmission elimination in 2014, these untreated LGAs needed to be reassessed with more sensitive tests to ascertain if there was transmission that needed to be addressed by an MDA program.

We conducted a survey in 2016 in southern Nigeria where the new onchocerciasis elimination program needed to determine if it should expand MDA to cover some or all of 19 suspected hypoendemic LGAs. One aspect of the 2016 survey was to determine the prevalence of high density of *Loa loa* mf (exceeding 30,000/mL of blood) using the new LoaScope technology.^14–17^ The *L. loa* portion of the study, which was reported by Emukah et al.,^18^ found that the 19 LGAs studied did not have high densities of loiasis that would preclude ivermectin MDA.^19,20^ The second aspect of the 2016 study was to determine the serological prevalence of Ov16 IgG4 antibodies to onchocerciasis, as well as a comparison between the two tools used: a rapid diagnostic test (RDT) and an ELISA. In this report, we provide those results and consider the various proposed Ov16 thresholds for launching MDA in hypoendemic areas.

## MATERIALS AND METHODS

### Study area.

The study area consisted of five states in the south–south and southeast geopolitical zones of Nigeria: Abia, Anambra, Delta, Ebonyi, and Imo states. This area is forested and features a 9-month rainy season and a 3-month dry season.^21^ The populace primarily makes its living through farming and fishing.^22^

### Study design.

The study was conceptualized to address two major concerns that limited progress toward onchocerciasis elimination in Nigeria: determining the safety of ivermectin treatment in *L. loa*–coendemic areas^14–17^ and determining hypoendemic LGAs in need of ivermectin treatment. Based on the aforementioned concerns, our selection of villages was purposive in an effort to find the places with hypoendemic onchocerciasis and, concurrently, loiasis infection with density levels that could be of concern. We identified LGAs in the five states that had never received ivermectin treatment but that likely harbored onchocerciasis transmission because of their close proximity to meso- or hyperendemic onchocerciasis LGAs. Within those LGAs, we used loiasis prevalence data collected between 2012 and 2015 by the FMOH and The Carter Center to select villages that appeared most likely to harbor high-density *L. loa* infection*.* Therefore, proximity to rivers was not a major factor in village sample selection.

Four blood tests were performed in the study. Two of these determined loiasis presence and intensity: the LoaScope test, and thin blood smears read by microscopy.^18^ The other two tests were to detect Ov16 antibodies: a relatively new commercial RDT, the SD BIOLINE Onchocerciasis IgG4 test (Standard Diagnostics, Gyeonggi-do, Republic of Korea),^23^ and the ELISA Ov16 methodology that was developed by the Onchocerciasis Elimination Program for the Americas (OEPA) (the “OEPA ELISA”) in 10% of samples.^24^ The study protocol was approved by the National Health Research Ethics Committee of Nigeria and Emory University’s Institutional Review Board in the United States.

### Procedures.

In 19 LGAs in five states, 110 villages were selected and a sample of 100 persons was sought in each village: 50 adults (older than of 18 years) and 50 children (aged 5–9 years). Before the study commenced in each village, the research team met with village leaders to obtain their verbal consent for the study to occur; these leaders were also responsible for selection of the survey venue for adults, typically a common meeting area. Town announcers summoned adults, resulting in a convenience sample. Village primary schools were also selected by convenience, but the children in the study were selected using systematic random sampling in those schools. Participants were all informed of the study purpose and procedures and provided written consent (adults) or assent (children). Persons were excluded from the study if they were ill or could not tolerate a finger stick for blood collection. Each participant received a unique identification (ID) number via a strip of barcode labels. The unique ID was captured electronically in tablets and affixed to the paper forms capturing demographic information and the samples for each diagnostic test. Each subject’s participation lasted about 10 minutes.

Consenting participants provided whole blood samples via standard finger-lancing between 10 am and 4 pm (when the microfilaria of *L. loa* circulate). Technicians wore gloves and cleaned the site with isopropyl alcohol before the finger stick. A sterile, single-use lancet was used on the third or fourth finger of each subject.

From each blood sample, one 10-µL capillary tube was immediately tested by using a Bioline Ov16 rapid test card. A special 15-µL capillary tube (Vitrocom, Mountain Lakes, NJ) was also immediately tested by using the LoaScope. Results of the RDTs and readings from the LoaScope were recorded in the aforementioned tablets and paper forms. A third 10-µL capillary was used in a subset of participants to make a thin smear blood slide for later examination for confirmation of LoaScope results; this was set aside to air-dry for later analysis in The Carter Center laboratory in Owerri.^18^

An additional 100 µL of additional blood was collected on a filter paper from 2,186 adults, for confirmation of RDT results by Ov16 ELISA. The adults in this group were all sampled residents of 42 villages randomly selected from among the study’s “first-line villages” (those located close to rivers), to increase the chance for positives to allow for comparison between the two tests. The dried blood spots (DBSs) were dried, stored in groups of 20 in plastic ziplock bags with a desiccant, and refrigerated. Of the blood spots collected, 1,000 were sent to PATH (who donated the RDTs for this study) in Seattle, Washington. The remaining 1,186 blood spots were analyzed at The Carter Center laboratory in Jos where they underwent serum elution and testing in the OEPA Ov16 ELISA, to compare with RDT results.

### Data analysis.

Electronic data stored in the tablets were transferred to MS Excel (using only ID number identifiers; Microsoft Corporation, Redmond, WA) to be cleaned and analyzed. The paper forms that contain the unique identifiers were stored securely at The Carter Center Owerri office. CIs comparing RDTs with ELISA were calculated with Epi Info 7 (OpenEpi), and SPSS 24 (IBM Corp., Armonk, NY) was used for other statistical analyses.

### Mass drug administration treatment thresholds and target LGAs and populations.

A number of different proposals have been made for Ov16 prevalence thresholds for launching MDA expansion into hypoendemic onchocerciasis areas. We used our RDT Ov16 results to evaluate six different thresholds, some of which have been entertained by the Nigeria Onchocerciasis Elimination Committee (NOEC) and/or the WHO Onchocerciasis Technical Advisory Subgroup (OTS): 1) an LGA average of ≥ 1% Ov16 positive children (NOEC 2017),^25^ 2) any village in an LGA with ≥ 1% Ov16 in children, 3) an LGA average of ≥ 2% in adults (NOEC 2019),^26^ 4) any village in an LGA with ≥ 2% in adults (OTS 2017),^27^ 5) an LGA average of ≥ 5% positive in adults, and 6) any village in an LGA with ≥ 5% in adults (OTS 2019).^28^ For each threshold, we calculated the number of LGAs and the treatment-eligible population that would need MDA for each of the six threshold proposals.

## RESULTS

The study teams visited 110 villages in 19 LGAs and tested 10,578 persons with the Ov16 RDT: 5,276 children (5–10 years, median age 8 years), and 5,302 adults (> 18 years, median age 45 years). Roughly half of the children (49.7%) who participated were female (2,624 of 5,276), and among adults, female participation was higher at 59.3% (3,142 of 5,302) (chi-square 92, *P* < 0.05). At least three villages were visited per LGA except in Onitsha North and Onitsha South, where each had only one village sampled.

The RDT results by LGA are shown in Table 1. Overall, there were 199 positives (1.9%). Adults were six times more likely to be positive for Ov16 antibodies than children (173/5,302 adults RDT positive [3.3%] versus 26/5,276 children [0.5%]). Among adults, village prevalence by RDT ranged from 0% to 58%. Among children, village RDT prevalence ranged from 0% to 4%. Five LGAs and 52 villages had no positive RDT results, but 14 (74%) of the LGAs and more than half (58/110) of sampled villages had at least one RDT-positive resident.

**Table 1:** Rapid diagnostic test results by LGA: total sample, adults, and children

LGA	Total sampled			Adults sampled			Children sampled		
	RDT positive	Total sampled	Proportion positive (%)	RDT positive	Total sampled	Proportion of adults positive (%)	RDT positive	Total sampled	Proportion of children positive (%)
Abakaliki	44	1,698	2.6	35	849	4.1	9	849	1.1
Anambra East	6	499	1.2	3	248	1.2	3	251	1.2
Anambra West	3	500	0.6	0	250	0.0	3	250	1.2
Ethiope East	7	965	0.7	6	489	1.2	1	476	0.2
Isoko North	5	500	1.0	4	249	1.6	1	251	0.4
Isoko South	9	500	1.8	3	249	1.2	6	251	2.4
Nkwerre	0	300	0.0	0	150	0.0	0	150	0.0
Ogbaru	5	997	0.5	4	498	0.8	1	499	0.2
Oguta	2	592	0.3	1	296	0.3	1	296	0.3
Ohaji–Egbema	0	200	0.0	0	101	0.0	0	99	0.0
Ohaukwu	108	1,053	10.3	107	558	19.2	1	495	0.2
Onitsha North	1	100	1.0	1	49	2.0	0	51	0.0
Onitsha South	0	100	0.0	0	49	0.0	0	51	0.0
Oru East	1	474	0.2	1	209	0.5	0	265	0.0
Oru West	0	258	0.0	0	129	0.0	0	129	0.0
Osisioma	0	365	0.0	0	181	0.0	0	184	0.0
Patani	3	592	0.5	3	297	1.0	0	295	0.0
Ughelli North	4	500	0.8	4	250	1.6	0	250	0.0
Ugwunagbo	1	385	0.3	1	201	0.5	0	184	0.0
Total	199	10,578	1.9	173	5,302	3.3	26	5,276	0.5

LGA = local government area; RDT = rapid diagnostic test.

Dried blood spots for in-parallel ELISA Ov16 testing were obtained from 2,186 subjects, but results are only available for the 1,186 DBSs that were analyzed by the laboratory in Jos (the 1,000 blood spots sent to PATH were not analyzed). There were 62 (5.2%) ELISA positives compared with 60 (5.1%) RDT positives. The difference between Ov16 RDT and ELISA results was not statistically significant (95% CI on RDT 3.8–6.3%, 95% CI on ELISA 4.0–6.5%, [*P* = 0.85]). Comparing Ov16 ELISA and RDT results by individual, we found 99.3% agreement overall (1,178 blood samples), with 91.9% agreement among positives (57 samples) and 99.7% agreement among negatives (1,121 samples). We had eight discordant results: three RDT positives were ELISA negative and five RDT negatives were ELISA positive.

### Mass drug administration treatment thresholds and resultant target LGAs and populations.

**Figure 1.**
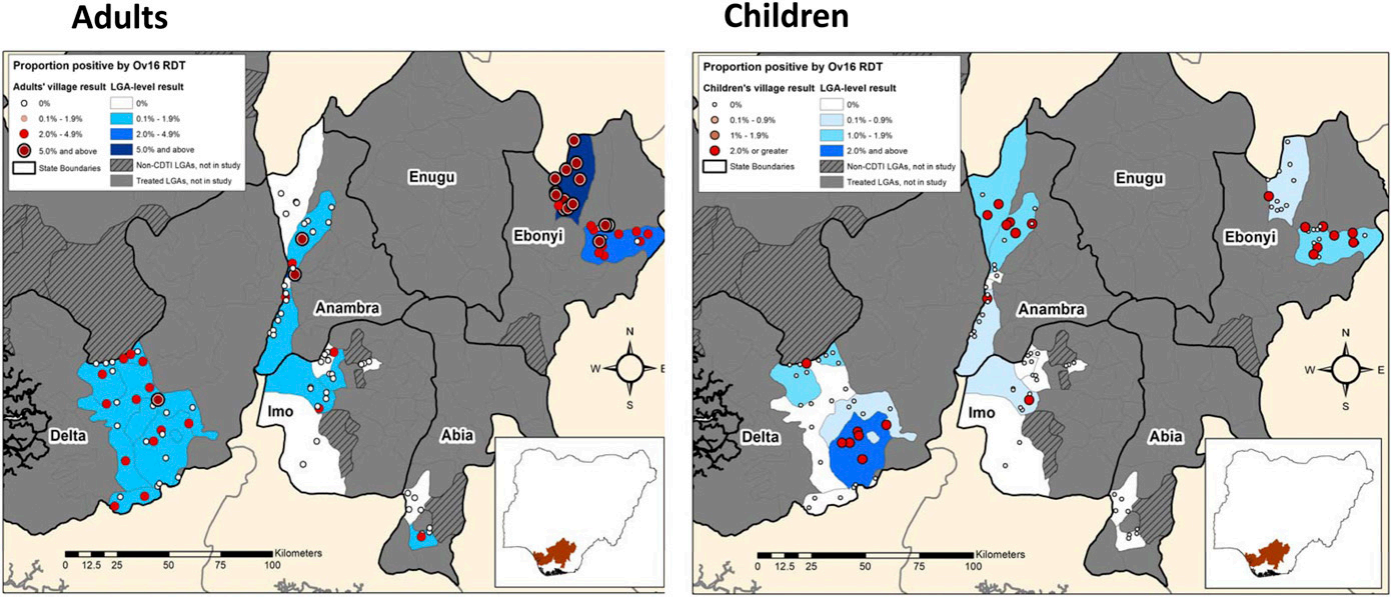
Hypoendemic onchocerciasis local government areas: Ov16 average prevalence in study villages by age group. This figure appears in color at www.ajtmh.org.

**Figure 2.**
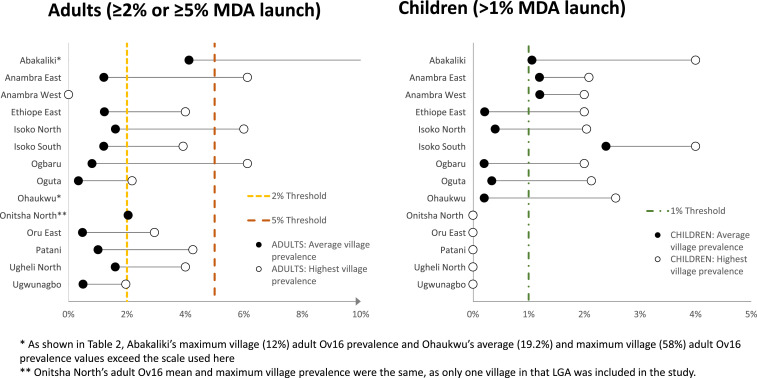
Mean vs. maximum village Ov16 rapid diagnostic test results for adults and children, by local government area (vertical lines show MDA launch thresholds). MDA = mass drug administration. This figure appears in color at www.ajtmh.org.

Figure 1 shows the Ov16 LGA results geographically, by adults and children, which provides a visual account of the six possible MDA decision thresholds. The study LGAs are shaded to reflect the mean prevalence results in three key MDA decision categories: 1% in children, 2% in adults, and 5% in adults. The dot map in the figure shows the positions of the sampled villages shaded to reflect their prevalence ranges. The pattern suggests that Ov16 prevalence decreases moving from north to south in this part of Nigeria. The range of these results is also shown graphically (with the thresholds indicated by lines) in Figure 2. Table 2 shows the treatment-eligible populations (calculated as 80% of the total population) in the 19 LGAs, together with the LGA average RDT prevalence and maximum village RDT prevalence in the total sample, adults, and children. The shaded cells in the table indicate when an MDA launch threshold was met. Note that although the sample from 50 children resulted in villages with one child positive having a 2% prevalence, the treatment decision would not have been different if the sample had been 100 with the same positive child (1%), and so did not influence the analysis.

**Table 2:** Local government area population and average vs. highest village rapid diagnostic test results by LGA: total sample, adults, and children (bold indicates a mass drug administration launch threshold was met)

LGA	80% Of population (treatment target if eligible)	Average of villages’ proportions positive (%)			Highest village proportion positive (%)		
		Total	Adults	Children	Total	Adults	Children
Abakaliki	171,505	2.6	**4.1**	**1.1**	12.0	**20.0**	**4.0**
Anambra East	173,322	1.2	1.2	**1.2**	3.0	**6.1**	**2.1**
Anambra West	189,244	0.6	0.0	**1.2**	1.0	0.0	**2.0**
Ethiope East	226,971	0.7	1.2	0.2	2.0	**4.0**	**2.0**
Isoko North	162,950	1.0	1.6	0.4	3.0	**6.0**	**2.0**
Isoko South	257,401	1.8	1.2	**2.4**	3.0	**3.9**	**4.0**
Nkwerre	90,602	0.0	0.0	0.0	0.0	0.0	0.0
Ogbaru	250,808	0.5	0.8	0.2	3.0	**6.1**	**2.0**
Oguta	161,653	0.3	0.4	0.4	1.0	**2.2**	**2.1**
Ohaji–Egbema	206,337	0.0	0.0	0.0	0.0	0.0	0.0
Ohaukwu	221,935	10.2	**19.2**	0.2	33.7	**58.0**	**2.6**
Onitsha North	141,232	1.0	**2.0**	0.0	1.0	**2.0**	0.0
Onitsha South	154,480	0.0	0.0	0.0	0.0	0.0	0.0
Oru East	126,401	0.1	0.5	0.0	0.8	**2.9**	0.0
Oru West	132,810	0.0	0.0	0.0	0.0	0.0	0.0
Osisioma	248,267	0.0	0.0	0.0	0.0	0.0	0.0
Patani	76,535	0.5	1.0	0.0	2.1	**4.3**	0.0
Ughelli North	362,884	0.8	1.6	0.0	2.0	**4.0**	0.0
Ugwunagbo	93,390	0.3	0.5	0.0	1.0	**2.0**	0.0
Total/mean positive	3,448,727	1.9	**3.3**	0.5	3.6	**6.4**	**1.2**

LGA = local government area.

Table 3 shows the populations that would be treated in each of the six MDA treatment decisions by LGA. The bottom line of Table 3 shows how consequential the selection of a prevalence threshold will be for the expansion of MDA into hypoendemic LGAs under the elimination paradigm. The decision resulting in the smallest target population was having an LGA average of ≥ 5% positive in adults (one LGA, 221,935 targeted), followed in order by an LGA average of ≥ 2% in adults (three LGAs, 534,672 targeted), an LGA average of ≥ 1% Ov16 positive children (four LGAs, 791,472 targeted), any village in an LGA with ≥ 5% in adults (five LGAs, 980,520 targeted), any village in an LGA with ≥ 1% Ov16 in children (nine LGAs, 1,815,789 targeted), and finally any village in an LGA with ≥ 2% in adults (13 LGAs, 2,426,987 targeted). It should be noted that the three lowest expansions (1–4 LGAs) resulted from the averaged analysis. Greatly increased target populations occurred when the threshold could be met by a single village value, and the 2017 OTS recommendation resulted in the largest expansion. No LGA met all six thresholds, but Ohaukwu and Abakaliki met five. Five (26%) of the 19 LGAs failed to meet any of the six MDA thresholds (Nkwerre, Ohaji–Egbema, Onitsha South, Oru West, and Osisioma). The combined eligible population of these five LGAs to be left untreated was 832,496.

**Table 3:** Mass drug administration decisions and population to be treated based on six different launch thresholds, by LGA

LGA	MDA launch thresholds					
	Mean	Maximum	Mean	Maximum	Mean	Maximum
	Adults ≥ 2%	Adults ≥ 2%	Adults ≥ 5%	Adults ≥ 5%	Kids ≥ 1%	Kids ≥ 1%
Abakaliki	171,505	171,505	–	171,505	171,505	171,505
Anambra East	–	173,322	–	173,322	173,322	173,322
Anambra West	–	–	–	–	189,244	189,244
Ethiope East	–	226,971	–	–	–	226,971
Isoko North	–	162,950	–	162,950	–	162,950
Isoko South	–	257,401	–	–	257,401	257,401
Nkwerre	–	–	–	–	–	–
Ogbaru	–	250,808	–	250,808	–	250,808
Oguta	–	161,653	–	–	–	161,653
Ohaji–Egbema	–	–	–	–	–	–
Ohaukwu	221,935	221,935	221,935	221,935	–	221,935
Onitsha North	141,232	141,232	–	–	–	–
Onitsha South	–	–	–	–	–	–
Oru East	–	126,401	–	–	–	–
Oru West	–	–	–	–	–	–
Osisioma	–	–	–	–	–	–
Patani	–	76,535	–	–	–	–
Ughelli North	–	362,884	–	–	–	–
Ugwunagbo	–	93,390	–	–	–	–
Total to be treated	534,672	2,426,987	221,935	980,520	791,472	1,815,789

LGA = local government area; MDA = mass drug administration.

## DISCUSSION

More than 10,500 adults and children in south Nigeria, residents of ivermectin-naive but potentially onchocerciasis-endemic villages, were tested by a commercial RDT for Ov16 IgG4 antibodies. The RDT results demonstrated a wide range of reactivity, with adult RDT village prevalence ranging from 0% to 58% among adults and from 0% to 4% among children. Adults were six times more likely than children to be Ov16 positive. Ov16 prevalence decreased geographically from north to south, with five of 19 LGAs and 52 of 110 villages having no RDT-positive individuals. The most likely reason for this is that in southern coastal Nigeria, slower flowing rivers and brackish waters are less favorable for the breeding of the blackfly vectors of onchocerciasis.

We conclude that these RDT results indicate that a part of the population studied ought to be placed under ivermectin MDA. However, the number of people (villages/LGAs) to be targeted for treatment varies enormously depending on the “launch MDA” Ov16 serological threshold selected. This is an area of considerable controversy among technical experts.

The NOEC was established in 2015 to provide independent guidance to help the FMOH reach its goal of onchocerciasis transmission elimination.^29^ Among its earliest recommendations, the NOEC outlined an approach to expand MDA into ivermectin-naive “hypoendemic” LGAs with ongoing transmission of the parasite.^30^ The original approach recommended by the NOEC to detect such areas was to assess children in high-risk villages (“first-line” villages located close to a riverine vector-breeding site) using Ov16 RDT or ELISA antibody testing, with a threshold for launching MDA being ≥ 1% seroprevalence (e.g., three positive children in 300). Children were chosen over adults as the indicator group because 1) Ov16 antibodies are detectable for years so in adults does not necessarily reflect recent infection,^31,32^ and 2) focusing on children is consistent with the WHO stop MDA guidelines.^33^ The NOEC opined that assessments in (more mobile) adults would not indicate recent or local transmission, given their more likely travel into nearby bordering LGAs known to have or have had onchocerciasis transmission. It should be noted here that two LGAs (Anambra West and Isoko South) in this study showed the surprising results of adults having roughly equal or lower Ov16 prevalence compared with children. We do not have any explanation for this finding other than it might be a chance occurrence in low-prevalence areas evaluated with small sample sizes.

In 2017, the WHO Geneva established an OTS to provide guidance needed by onchocerciasis elimination programs. One area of OTS focus has been the approach to rapid assessment in ivermectin-naive areas to detect active transmission, termed “onchocerciasis elimination mapping” (OEM). The OTS has discussed OEM indicator age groups (adults versus children), sampling strategies (first-line, high-risk villages versus random selection), Ov16 diagnostic platforms (ELISA or RDT), and “launch MDA” thresholds. In contrast to the NOEC’s recommendations, the OTS chose adults as the indicator group with the reasoning that 1) it is essential for an elimination program not to miss transmission areas, 2) adults will have higher Ov16 antibody prevalence than children, and thus, 3) compared with children, adults would give a more reliable (sensitive) Ov16 signal in small samples. A ≥ 2% Ov16 threshold in any sampled village was recommended as the “launch MDA” threshold for a district. The 2019 OTS meeting changed its recommendations to ≥ 5% in adults in any sampled village in a district.^28^ The OTS proposed a two-step sampling procedure of purposeful survey (first-line, high-risk villages) followed by random sampling if the results from first-line village testing is negative. The second stage is being examined in ongoing operational research, and to our knowledge, this has not yet been operationally implemented by any national program at the time of this publication. The OTS recommendations from its 2017 and 2018 meetings have been published in the WHO *Weekly Epidemiological Record*.^27,34^

In 2018, the NOEC revised its OEM approach to be consistent with the OTS recommendation, except that it continued to recommend the use an LGA average of ≥ 2% in adults for a launch MDA decision for an LGA, rather than the OTS recommendation of ≥ 2% in any sampled village in that LGA. Ultimately, resolution of the OEM debate will depend on correlating rapid assessments in the human populations with corresponding entomological indicators of transmission, such as the presence and density of *Simulium* vectors, vector infection and infectivity, and calculation of transmission potentials.

The eagerness to be certain to eliminate all onchocerciasis transmission must be tempered with the real limitations of funding available for national programs, both in terms of the costs of the OEM sampling approach required and the cost implications resulting from the selection of the MDA threshold. In regard to the latter consideration, in this report, we share our results from an MDA threshold exercise that produced recommendations ranging from treating just one additional LGA (221,935 persons eligible for treatment) to treating 13 additional LGAs (2,426,987 persons). The threshold resulting in the largest numbers was from the 2017 OTS recommendation for MDA in a region where any village has ≥ 2% Ov16 positivity in adults. If twice-per-year ivermectin treatment was elected in ivermectin-naive hypoendemic areas according to African Programme for Onchocerciasis Control recommendations,^35^ this “cast a broad net” approach would require 4.5 million treatments per year in our study area.

Based on parallel testing of 1,186 persons, we found that the RDTs performed in the field were not inferior to ELISA laboratory testing on DBSs. The positivity rates were similar (5.1% RDT versus 5.2% ELISA), and there was a 91.9% agreement among positives. However, the WHO OTS has been concerned with results from other studies showing poor performance of the RDT in low-prevalence areas and recommends against using the RDT without an ELISA DBS backup.^36^ We suggest that the recognized variability of RDT performance may be due to lot-to-lot variation in RDT quality and suggest that a positive control be supplied with each shipment so that quality control can be assured in the country where the tests are to be used.

This study had several limitations. First, the approach we used to select our village samples was designed primarily to identify high-risk *L. loa* areas; villages were selected in known *L. loa* areas^37^ rather than in first-line, high–onchocerciasis risk villages as recommended both by the OTS and NOEC for OEM,^27,38^ although we note that some first-line villages were included in the villages sampled (and all of the villages where DBSs were obtained for the ELISA evaluation were first-line). Second, our study predates the NOEC and OTS OEM recommendations that call for a sample size of 100 persons *in a given age group* per village.^27,39^ Our sample from 100 persons included 50 adults and 50 children per village. The ideal comparative OEM study would have had 100 adults and 100 children per village. The impact of this was especially important in the analysis of the “≥ 1% Ov16 positive children in any village” threshold category. Given the sample from 50 children, a single Ov16 positive child resulted in a 2% prevalence. However, if we had taken a sample from 100 children, the same single positive child would have been sufficient to meet the ≥ 1% threshold MDA value, so this limitation did not bias our results or conclusions. Third, as already noted, our sample was one of convenience: in each village, adults voluntarily reported to a central village location, and children were sampled in one selected school. A random sample would have reduced the risk of biases influencing the results. Fourth, we only tested a little more than half of the subsample for the Ov16 ELISA comparison. This half came from a randomly selected sample of villages from the roster of first-line villages. We are assuming that the results from this subsample do not differ from those DBSs left untested, but this cannot be proven either way.

## CONCLUSION

In this study, the Ov16 RDT functioned well as a discriminatory diagnostic tool in communities and districts (LGAs) in onchocerciasis-hypoendemic areas, providing results consistent with the expected epidemiology of the infection in southern Nigeria. There was no statistical difference between field-based RDT results and laboratory Ov16 ELISA testing (using the OEPA methodology) of a subsample of DBSs. In ivermectin-naive areas, there is an urgent need for comparative entomological assessments to determine the Ov16 serological thresholds that reflect where onchocerciasis transmission is ongoing and ivermectin MDA is truly needed.
